# Collargol: A Review of Some of Its Clinical Applications, with Experiments on Its Antiseptic Action

**Published:** 1903-12

**Authors:** J. M. Fortescue-Brickdale

**Affiliations:** Registrar and Pathologist to the Royal Hospital for Sick Children and Women, Bristol. (From the Bacteriological Research Laboratory, University College, Bristol.)


### COLLARGOL: A REVIEW OF SOME OF ITS CLINICAL APPLICATIONS, WITH EXPERIMENTS ON ITS ANTISEPTIC ACTION.

BY

J. M. Fortescue-Brickdale, M.A., M.D. Oxon.,

*Registrar and Pathologist to the Royal Hospital for Sick Children and Women, Bristol.*

*(From the Bacteriological Research Laboratory, University College, Bristol.)*

The purpose of the present article is to review and criticise the results obtained by the internal use of silver in diseases which may broadly be said to belong to the group of bacterial intoxications. The cases hereafter to be quoted will show in what a variety of conditions this drug has been employed, but the underlying indication has in all been the presence, either proved or assumed, of bacteria and their products in the system. Silver as an antiseptic was brought into prominence by Credé of Dresden, who in 1897 made a communication on the subject to the International Congress of Medicine at Moscow. He first used salts of silver (citrate and lactate) as wound dressings, and later employed the substance known as collargol, which he thought had a direct antiseptic action in internal diseases. I propose now to give a short account of the physical and physiological properties of this substance, then a description of the mode of its application, a summary of the clinical results, and lastly an account of the experimental evidence as to its utility.

**Physical Properties.**—Collargol (an allotropic modification of silver) is a blackish brittle substance with a slight metallic lustre, capable of forming an opaque black liquid when rubbed up with distilled water up to a proportion of 1 in 25. Weaker solutions are light brown in colour, and all leave an irridescent film on drying. It is not very stable, but is said to keep fairly well in the dark, especially if a little albumin is added to the "solution."

**Physiological Properties.**—Brunner states that a leucocytosis is established six hours after an intravenous injection, which attains its maximum in twenty-four hours, and has disappeared at the end of two days. Beyer states that in rabbits (after the injection of large doses) collargol may be found in the blood in eight to ten hours; he also found it in various organs eight or nine days later. It was excreted mainly by the intestine, and although he did not find it in the kidney, he considers that it is also eliminated in that way. Blood retains the colloid silver in solution remarkably well, and prevents its precipitation or conversion into the ordinary metallic form. All observers agree that it is rapidly eliminated, and that no poisonous symptoms ever occur.

**Methods of Application.**—Collargol has been employed in human and veterinary medicine. For internal use in infective diseases two forms are prepared: (1) An ointment containing 15 per cent. of the drug, a few grams of which are rubbed into the carefully cleaned healthy skin once a day or oftener; (2) A solution to be freshly prepared, as required, from the solid substance for intravenous injection. The strength of this solution is usually 1 per cent., 5 or 10 cc. (sometimes more) being injected once a day, or at intervals of two or three days.

**Clinical Results.**—I have been able to collect forty-four cases from the literature in which a clinical account, sufficiently full for critical purposes, is given. I shall divide them into five classes, for practical purposes, as follows :—

I. Cases in which other treatment was given which may fairly be credited with the results (six cases) :—

1. NETTER. Rheumatic pericarditis with recovery. Inunction + salicylates.

2. BRAINERD. A case diagnosed as general tuberculosis, with subacute symptoms suggestive of meningitis. Improvement began after one month. Inunctions + very careful hygiene, tonics, etc. A choroidal tubercle vanished during treatment.

3. FISCHER. Anthrax. Injections + usual surgical treatment of pustule. Recovery.

4. MÜLLER. Old standing tuberculous knee, clearing up quickly under injections. Although not mentioned, accessory treatment is certain, as patient was removed from bad surroundings into hospital.

5. NETTER. Cerebro-spinal meningitis. Lumbar puncture + inunctions.

6. DVORETZKI. Furunculosis. Inunctions + usual surgical treatment.

II. Cases in which a doubt exists as to the diagnosis (four cases) :—

1. NETTER. Broncho-pneumonia, which cleared up (pneumococcal). Phthisis subsequently diagnosed (although tubercle bacilli were never found) owing to signs of cavitation at one apex.

2. MEIER. Case operated on for mastoid disease. After operation temperature remained high, but fell in three days (inunctions). Lateral sinus thrombosis was diagnosed.

3. HAASE. Amputation with post operative fever. Stump appeared healthy. Temperature fell after one injection. Septicæmia diagnosed.

4. CREDÉ. Fractured pelvis and other injuries. Eight weeks after injury rigors occurred, which ceased after two injections. Septic peritonitis diagnosed.

III. Cases in which the influence of collargol is, from the nature of the case, doubtful (five cases) :—

1. NETTER. Pneumococcal pneumonia. Crisis on sixth day (inunctions).

2. NETTER. Enteric in a child (no "widal" done). Severe initial symptoms. Temperature normal in nineteen days (inunctions).

3. DVORETZKI. Erysipelas. Recovery after three inunctions.

4. CREDÉ. An advanced case of phthisis, in which (apparently during one week only) improvement occurred after injections.

5. NETTER. Scarlet fever. A severe case with recovery (inunctions).

IV. Cases in which collargol was inert (eighteen cases) :—

1–6. STROHMAYER. Puerperal fever. Three died of pyæmia Two recovered under energetic surgical treatment.

No effect observed after inunctions. One case in which temperature rose after the inunctions were discontinued, though they had no marked effect otherwise. Death from general peritonitis.

7–8. STROHMAYER. Streptococcal empyema.

9. STROHMAYER. Glandular abscesses after scarlet.

10. STROHMAYER. Furunculosis (yielded to incisions easily).

11. STROHMAYER. Popliteal abscess. Amputation subsequently necessary.

12. STROHMAYER. Middle ear suppuration after scarlet fever. Death.

13–18. NETTER. Tuberculous meningitis. (These cases are not reported, but Netter states that inunctions were inert.)

V. Cases favourably influenced by collargol (seventeen cases):—

1–3. S. S. JONES. Septic endometritis; POLAK. Septic endometritis; HEINSHEIMER. Parametritis. These cases occurred after delivery or abortion. The general (toxæmic) symptoms were severe. Adequate stimulant treatment, etc. In Jones' case only was local treatment discontinued when inunctions were begun. In all the inunctions were frequent at first (more frequent than in Strohmayer's cases), and improvement rapid. In none was there direct evidence of general septicæmia or pyæmia.

4. WITTHAUER. Empyema. Complete aspiration impossible. Rapid recovery under inunctions (12 days).

5–9. DVORETZKI and WERLER. Furunculosis. In four local treatment failed till inunctions were begun. In one local treatment not mentioned.

10–12. KLOTZ and WENKELBACH. In these symptoms certainly pointed to septic endocarditis. A fall of temperature and general improvement followed a few days' treatment with injections. S. pyogenes albus said to have been isolated from blood in one case, but this organism is practically always due to a contamination of the culture.

13. NETTER. Perinephric abscess. Operation. Wound healed five days after removal of tube.

14. NIESSEN. Chronic arthritis. A case which had resisted treatment for some time. No more attacks of pain occurred after twenty daily inunctions. A peculiar organism isolated from blood, but not proved to be ætiological.

15. DVORETZKI. Osteomyelitis. Six months' history of pain and discharge. Complicated by enteritis. Improvement in general condition after a week (inunctions).

16. CREDÉ. Acute rheumatism, with pleuritic effusion and pericarditis. Ordinary treatment had failed, but improvement occurred after two injections (48 hours). Patient then got worse, but after three injections (10 days) convalescence was established.

17. CREDÉ. Extensive gangrene. Incisions had been made. General symptoms improved, and temperature fell after each injection.

For critical purposes the first fifteen cases (Classes I.–III.) are obviously useless. In the remainder, seventeen are favourable to the efficiency of collargol and eighteen (including Netter's six cases, which are unreported, but being negative are admissible) are unfavourable. It seems clear that in about half the cases, therefore, collargol had an influence—principally seen in a reduction of temperature. In no case has it been proved to have influenced a general pyæmic or septicæmic condition, except in one case of Wenkelbach's, in which there were emboli which may have had a septic origin. In the pyæmic case recorded by Strohmayer, in which the temperature rose after the inunctions were discontinued, no amelioration of the symptoms had occurred during their application.

**Experimental Evidence.**—Credé in Germany and Bolton and Brown in America have proved that various metals have an inhibitory action on the growth of bacterio *in vitro*. The experiments of the two last-named observers are remarkably exact and very fully reported. Credé has classified the metals according to their inhibitory power, silver standing fourth on his list. The method adopted is to place a piece of metal on the surface of an inoculated Petri dish and observe the ring round it, on which few or no colonies appear. Credé states that a piece of silver placed on a septic wound is gradually absorbed, whereas this does not occur on an aseptic wound. I have only been able to find two accounts of animal experiments. Beyer inoculated two rabbits with anthrax, and injected one subcutaneously and the other intravenously with a large dose of collargol. Both recovered, but he makes no mention of a control animal. Trommsdorff calculated the dose of collargol for rabbits at ·0025–·0059 gm. He injected twenty-one rabbits in all, using the organisms of swine erysipelas and swine fever. The injections were given once or twice daily, and were begun in different animals either immediately or six, twelve, twenty-four aud thirty-six hours after inoculation. Ten animals received ten times the amount of collargol their weight assigned to them.

The animals weighed on an average 2,500 gms. Control animals were used, and his results in every case were negative.

I have been unable to find details of any test tube experiments as to the actual germicidal power of collargol, though it is stated to have inhibited S. pyogenes aureus, a fairly resistant organism, in solutions of ·05 per cent. to ·01 per cent. With a view to testing the effect of collargol as an antiseptic, I have been allowed to carry out the following experiments at the Bacteriological Laboratory of University College by the kindness of Professor Stanley Kent:—

A stock solution (50 per cent.) was prepared according to Credé's directions, and carefully kept in a stoppered bottle in the dark at room temperature. The cultivations of bacteria were all of twenty-four hours' growth, except those of anthrax, which were broth cultivations of 16–18 hours' growth. This practically prevented the formation of spores. Credé states that collargol has more effect on less virulent strains of cocci. It is to be noted, therefore, that my cultivations were prepared from ordinary laboratory strains of no especial virulence. Broth tubes were prepared containing 4 cc. each, to which 1 cc. of solutions of collargol was added, the strength of the latter being varied to bring the total to the required point. These broth and collargol tubes were then inoculated with the organisms, incubated at 37° C. for twenty-four hours and examined. The stronger solutions were so opaque that no growth (if present) could be seen. Coverslip preparations were therefore necessary; in weaker solutions these were also made to test the purity of the growth. The results were as follows:—

1 per cent. collargol. No growth of B. anthracis, S. pyogenes aureus, or strep. pyogenes.

·1 per cent. collargol. No growth of strep. pyogenes. Occasionally slight growth of B. anthracis and S. pyogenes aureus.

·05 per cent. collargol. No growth of strep. pyogenes. Growth of B. anthracis and S. pyogenes aureus.

·03 per cent. collargol. No growth of strep. pyogenes.

Growth of B. anthracis and S. pyogenes aureus (except in one case).

·02 per cent. and ·01 per cent. collargol. Growth of all three organisms.

Over seventy tubes were inoculated in this series. In addition to this, four glycerine Agar plates were prepared and inoculated with a mixed cultivation of streptococcus pyogenes and staphylococcus pyogenes aureus. After twenty-four hours' growth at 37° C., a solution of collargol was poured over the surface of the plate, and further incubation for twenty-four hours at 37° C. allowed. At the end of this time the surface of the Agar had become softened, and from the supernatant liquid broth tubes were inoculated and incubated in the same manner. The results with different strengths of collargol were:—

| | | | | |
|---|---|---|---|---|
| 1 | per cent. | collargol, | no growth. | |
| ·5 | ,, | ,, | growth of both | organisms. |
| ·1 | ,, | ,, | ,, | ,, |
| ·05 | ,, | ,, | ,, | ,, |

In solutions, therefore, of ·5 per cent. and under, collargol has no bactericidal effect on strep. pyogenes or S. pyogenes aureus after twenty-four hours' exposure; both these organisms grow freely in solutions of ·02 per cent., and B. anthracis will always grow in a ·05 per cent. solution.

In living blood plasma the inhibitory and bactericidal power of collargol is probably less than in a broth cultivation medium. Behring has shown that the power of all antiseptics varies inversely with the amount of albumin in the medium in which they act; and Credé, as noted above, states that metals inhibit virulent cultivations less readily than non-virulent ones. With regard to the amount of collargol in solution in the blood, only an approximate estimate can be made, and that only in the case of intravenous injections. The right ventricle in an average man contains about 100 cc.; the weight of silver, therefore, carried into it with each pulse wave represents the percentage solution it will pump into the pulmonary circulation. It is possible to inject 1 cc. of solution per pulse beat, and this in most cases would represent ·01 gm. collargol. The percentage therefore pumped out by the right ventricle would be ·01 per cent. At the outset of its course through the body, therefore, the strength of collargol would certainly be insufficient to inhibit even a delicate organism such as streptococcus pyogenes.

The general conclusions which may therefore be drawn as to the action of collargol are as follows:—

(1) That collargol has never been introduced into the body in sufficiently large quantities to produce an antiseptic solution in the mass of the blood.

(2) That the evidence is strongly against its having any effect on artificial septicæmia in rabbits.

(3) That as far as the above cases show it has not had any effect in proved cases of general pyæmia or septicæmia in man.

(4) That a fall of temperature and improvement in the general condition comparable to that sometimes produced by antipyretics and hydrotherapy has been observed in some cases of general toxæmia, but that in half the recorded cases admissible as evidence it proved inert.

### REFERENCES.

Credé.—*Berl. klin. Wchnschr.*, 1901, xxxviii. 941.
Beyer.—*München. med. Wchnschr.*, 1902, xlix. 331.
Brunner.—Quoted by Beyer.
Netter.—*Bull. Soc. méd d. Hôp.*, Dec., 1902.
Brainerd.—*South Calif. Pract.*, August, 1900.
Fischer.—*München. med. Wchnschr.*, 1901, xlviii. 1879.
Müller.—*Berl. klin. Wchnschr.*, March 13th, 1902.
Dvoretzki.—*Therap. Monatshr.*, 1900, No. 3.
Meier.—*München. med. Wchnschr.*, 1899, xlvi. 1411.
Haase.—*Ibid.*, 1902, xlix. 331 (discussion).
Strohmayer.—*Ibid.*, 1900, xlvii. 1064.
S. S. Jones.—*Obstetrics*, Feb. 2nd, 1899.
Polak.—*Post-Graduate*, 1900, xv. 475.
Heinsheimer.—*Allg. med. Centr.-Ztg.*, 1900, No. 49.
Witthauer.—*Diakonissen Anstalt. Halle a. S.*, 1900.
Klotz.—*Deutsche med. Wchnschr.*, 1902, 524.
Wenkelbach.—*Schmidt's Jarhb.*, cclxxii. 162.
Niessen.—*Wien. med. Wchnschr.*, 1899, Nos. 44 and 45.
Bolton.—*Tr. Assoc. Am. Physicians*, 1894, ix. 174.
Bolton & Brown.—*Ibid.*, 1897, xii. 488.
Trommsdorff.—*München. med. Wchnschr*, 1902, xlix. 1300.

